# Long non-coding RNA *Irm* enhances myogenic differentiation by interacting with MEF2D

**DOI:** 10.1038/s41419-019-1399-2

**Published:** 2019-02-21

**Authors:** Yutong Sui, Yu Han, Xingyu Zhao, Dongsong Li, Guangyu Li

**Affiliations:** 1 State Key Laboratory for Molecular Biology of Special Economic Animals, Institute of Special Animal and Plant Sciences, Chinese Academy of Agricultural Sciences, Changchun, 130112 China; 2 Joint Surgery Department, No.1 Hospital of Jilin University, Changchun, 130021 China

## Abstract

Recent studies suggest important roles for long non-coding RNAs as essential regulators of myogenic differentiation. Here, we report that lncRNA *Irm* is upregulated during myogenesis. Functional analyses show that the overexpression of *Irm* enhances myogenic differentiation, whereas the inhibition of *Irm* has completely opposite effects in vitro. Notably, the inhibition of *Irm* blocks damage-induced muscle regeneration in vivo. Mechanistically, *Irm* regulates the expression of myogenic genes by directly binding to MEF2D, which in turn promotes the assembly of MyoD/MEF2D on the regulatory elements of target genes. Collectively, we have identified a novel lncRNA that interacts with MEF2D to regulate myogenesis.

## Introduction

Long non-coding RNAs (lncRNAs) are a class of transcripts of more than 200 nucleotides without protein-coding potential. Within the last few years, thousands of lncRNAs have been identified and shown to play pivotal roles in numerous important biological processes, including cellular proliferation^1^, differentiation^2^ and development^3^, chromosomal imprinting^4^, and genomic stability^5^. Worth to note, several lncRNAs have been determined to regulate myogenesis. For example, lncRNA *AK017368* promotes the proliferation and suppresses the differentiation of myoblasts in skeletal muscle development by attenuating the function of miR-30c^6^. lncRNA *Dum* interacts with Dnmts to regulate Dppa2 expression during myogenic differentiation and muscle regeneration^7^. *Lnc-mg* promotes myogenesis, by functioning as a competing endogenous RNA for microRNA-125b to control the protein abundance of insulin-like growth factor 2^8^. However, the amount of characterized lncRNAs that regulate myogenesis is merely the tip of the iceberg, and a large number of lncRNAs remain to be characterized.

Myogenesis is a highly coordinated developmental process that contributes to the formation and maintenance of muscle tissue. Myogenic cell specification and differentiation are controlled by a complex network of myogenic regulatory factors, including MyoD (myogenic differentiation), muscle bHLH proteins Myf5, myogenin (MyoG), and MEF2 family^9–11^. Recent studies have indicated various molecular mechanisms for lncRNAs and the current best characterized is in the regulation of epigenetic dynamics and gene expression^12^. Indeed, some muscle-specific lncRNAs that control muscle gene expression have been reported, including lncRNA *Dum*^7^, muscle-specific linc-MD1^13^, *linc-RAM*^14^, and *Linc-YY1*^15^. lncRNA *Irm* has been previously identified as an alternatively splicing isoform of *Rian* gene^16^. Worth to note, a recent study has suggested that *Irm* was associated with the morphogenesis of skeletal muscle during embryonic development, indicating its pivotal role in myogenesis^17^. However, the biological function of *Irm* in the development of skeletal muscle remains unclear.

Here, we examined the functional role of *Irm* in the development of skeletal muscle. We showed that the expression of *Irm* is tightly associated with myogenic processes in vitro and in vivo. Furthermore, functional studies demonstrated that it acts as a pro-myogenic factor in both myoblast differentiation and muscle regeneration. Mechanistically, we revealed that *Irm* regulates the transcription of myogenic genes by directly binding to MEF2D, which in turn promotes the assembly of the MyoD–MEF2D complex on the regulatory elements of target genes.

## Results

### LncRNA *Irm* is associated with skeletal myogenesis

**Fig. 1:**
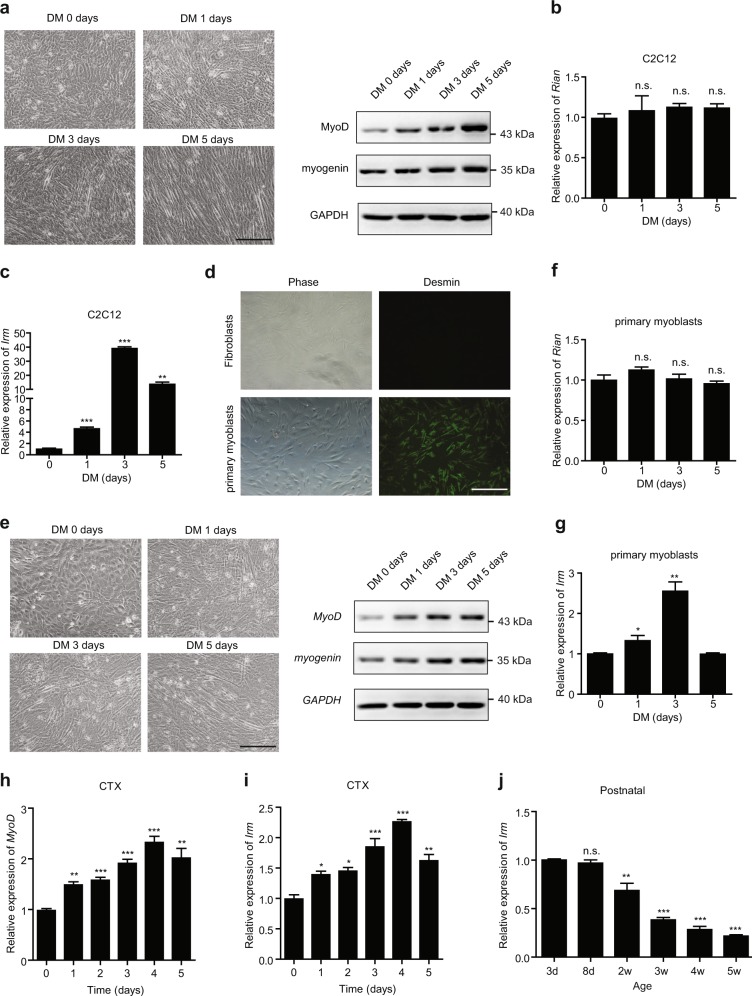
*Irm* is a myogenesis relevant lncRNA. **a** Left: the representative pictures of C2C12 cells at 0, 1, 3, and 5 days in differentiation medium; right: the protein levels of MyoD and myogenin were detected by western blotting in C2C12 cells at 0, 1, 3, and 5 days in DM. Scale bars, 50 mm. **b,** **c** The expression levels of *Rian* (**b**) and *Irm* (**c**) were detected by qRT-PCR in C2C12 cells at 0, 1, 3, and 5 days in DM. **d** The certification of the primary myoblasts freshly isolated from mouse limb muscles. Scale bars, 50 mm. **e** Left: the representative pictures of the primary myoblasts at 0, 1, 3, and 5 days in DM; right: the protein levels of MyoD and myogenin were detected by western blotting in primary myoblasts at 0, 1, 3, and 5 days in DM. Scale bars, 50 mm. **f,** **g** The expression levels of *Rian* (**f**) and *Irm* (**g**) were detected by qRT-PCR in primary myoblasts at 0, 1, 3, and 5 days in DM. **h,** **i** The mRNA levels of MyoD (**h**) and *Irm* (**i**) were detected by qRT-PCR during CTX-induced regeneration. **j** The expression levels of *Irm* were detected by qRT-PCR in muscles of postnatal mice. Data shown represent the mean ± SEM of three independent experiments. n.s., not significant; ^*^P < 0.05, ^**^P < 0.05, and ^***^P < 0.05 by Student’s *t* test. CTX, cardiotoxin; DM, differentiation medium; qRT-PCR, quantitative real-time polymerase chain reaction

Recent studies have shown that *Irm* is associated with the morphogenesis of skeletal muscle during embryonic development^17^. Therefore, we hypothesized that *Irm* may also be involved in myogenesis. To investigate its relevancy in myogenesis, we examined its temporal and spatial expression patterns in several myogenesis systems in vitro and in vivo. First, the C2C12 cells were shifted to Dulbecco's modified Eagle's medium (DMEM) containing 2% horse serum for myogenic differentiation experiment (Fig. 1a). We found that the expression of MyoD and myogenin was significantly increased during the differentiation of C2C12 cells (Fig. 1a). Meanwhile, the expression of *Rian* had no change during the differentiation of C2C12 cells (Fig. 1b). However, *Irm* was found to be significantly upregulated during the stage from day 0 to day 3 in the differentiation medium but gradually decreased afterwards (day 5) (Fig. 1c), suggesting that it can be a myogenic factor during differentiation. Furthermore, the primary myoblasts, which were isolated from 10-day-old mouse muscles, were shifted to the differentiation medium for myogenic differentiation experiment (Fig. 1d, e). Consistently, the expression of MyoD and myogenin was significantly increased during primary myoblast differentiation. Meanwhile, the kinetics of *Rian* and *Irm* expression was also confirmed during the differentiation of freshly isolated primary myoblasts (Fig. 1f, g). In addition, we examined the expression dynamics of *Irm* during myogenesis in vivo. By employing a cardiotoxin (CTX)-induced muscle regeneration model, we found that *Irm* is highly induced during the regeneration stage (Fig. 1h, i). Consistently, high levels of *Irm* were observed in the limb muscles of newborn mice (at the age of 3 days and 8 days), which displayed active myogenesis, but the level of *Irm* decreased as the neonatal myogenesis ceased after about 2 weeks and remained low as the mice aged (Fig. 1j). These results indicated that *Irm* is associated with active myogenesis in vitro and in vivo.

### *Irm* promotes myogenic differentiation

**Fig. 2:**
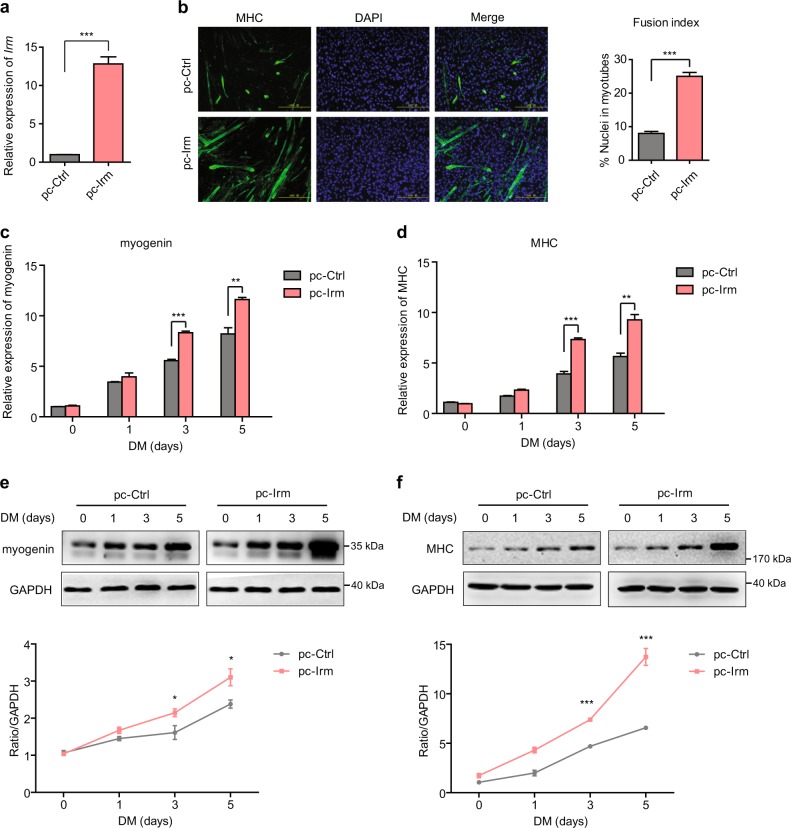
Overexpression of *Irm* enhances myogenic differentiation. **a** Relative expression levels of *Irm* in C2C12 cells expressing pcDNA3.1 (pc-Ctrl) or pcDNA3.1-*Irm* (pc-Irm), as detected by qRT-PCR. **b** The differentiation of *Irm*-overexpressed C2C12 cells was detected by staining for MHC at 48 h in DM. Fusion index was calculated. Scale bars, 50 mm. **c,** **d** The mRNA levels of myogenin (**c**) and MHC (**d**) were detected by qRT-PCR in *Irm*-overexpressed C2C12 cells at 0, 1, 3, and 5 days in DM. **e,** **f** The protein levels of myogenin (**e**) and MHC (**f**) were detected by western blotting in *Irm*-overexpressed C2C12 cells at 0, 1, 3, and 5 days in DM. Myogenin protein levels were normalized to the GAPDH protein levels. Data shown represent the mean ± SEM of three independent experiments. ^*^P < 0.05, ^**^P < 0.05, and ^***^P < 0.05 by Student’s *t* test. DM, differentiation medium; MHC, myosin heavy chain; qRT-PCR, quantitative real-time polymerase chain reaction

**Fig. 3:**
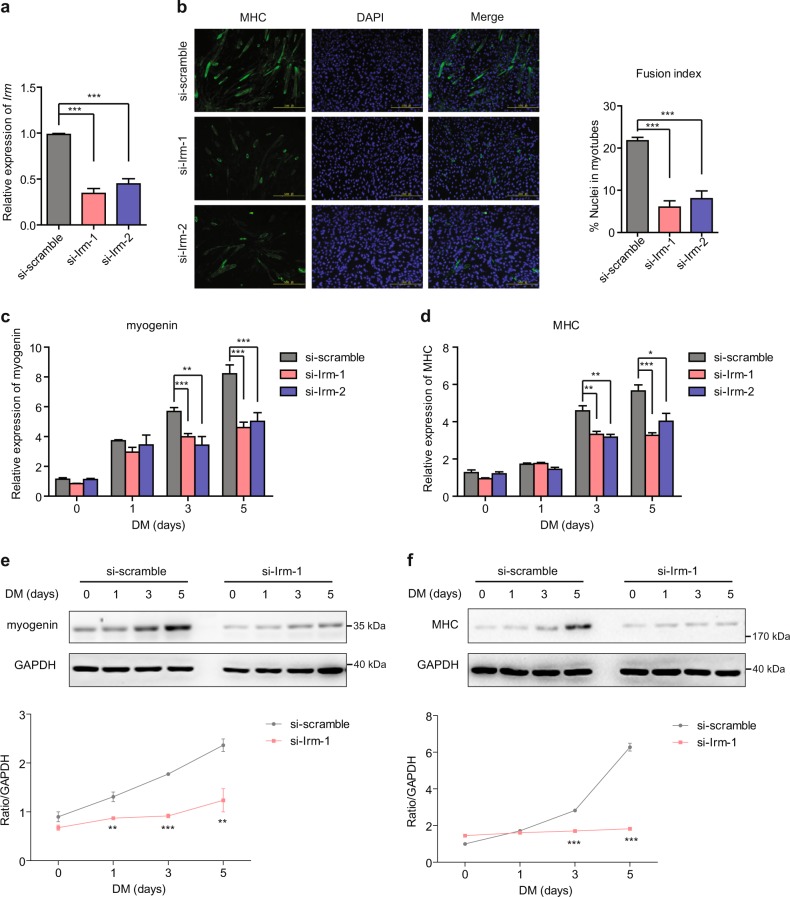
Knockdown of *Irm* inhibits myogenic differentiation. **a** Relative expression levels of *Irm* in C2C12 cells expressing si-scramble or si-Irm, as detected by qRT-PCR. **b** The differentiation of *Irm*-knockdown C2C12 cells was detected by staining for MHC at 96 h in DM. Fusion index was calculated. Scale bars, 50 mm. **c,** **d** The mRNA levels of myogenin (**c**) and MHC (**d**) were detected by qRT-PCR in *Irm*-knockdown C2C12 cells at 0, 1, 3, and 5 days in DM. **e,** **f** The protein levels of myogenin (**e**) and MHC (**f**) were detected by western blotting in *Irm*-knockdown C2C12 cells at 0, 1, 3, and 5 days in DM. Myogenin protein levels were normalized to GAPDH protein levels. Data shown represent the mean ± SEM of three independent experiments. ^*^P < 0.05, ^**^P < 0.05, and ^***^P < 0.05 by Student’s *t* test. DM, differentiation medium; MHC, myosin heavy chain; qRT-PCR, quantitative real-time polymerase chain reaction

Given the induction of *Irm* expression during C2C12 differentiation, it was conceivable that *Irm* plays a regulatory role in regulating myogenesis. Thus, we first examined the effect of *Irm* on the myogenic differentiation of C2C12 cells (Fig. 2a). The overexpression of *Irm* resulted in a significant increase in C2C12 cell differentiation as proved by the increased myosin heavy chain (MHC) immunostaining (Fig. 2b), increased fusion index (Fig. 2b), and the upregulated expression of myogenic marker genes myogenin and MHC (Fig. 2c–f). Conversely, the knockdown of *Irm* by two independent small interfering RNAs (siRNAs) (Fig. 3a) inhibited the differentiation of C2C12 cells with reduced MHC immunostaining (Fig. 3b), decreased fusion index (Fig. 3b), and downregulated myogenin and MHC expression (Fig. 3c–f). Taken together, all these results indicated that *Irm* promotes myogenic differentiation.

### Loss of *Irm* delayed CTX-induced muscle regeneration in vivo

**Fig. 4:**
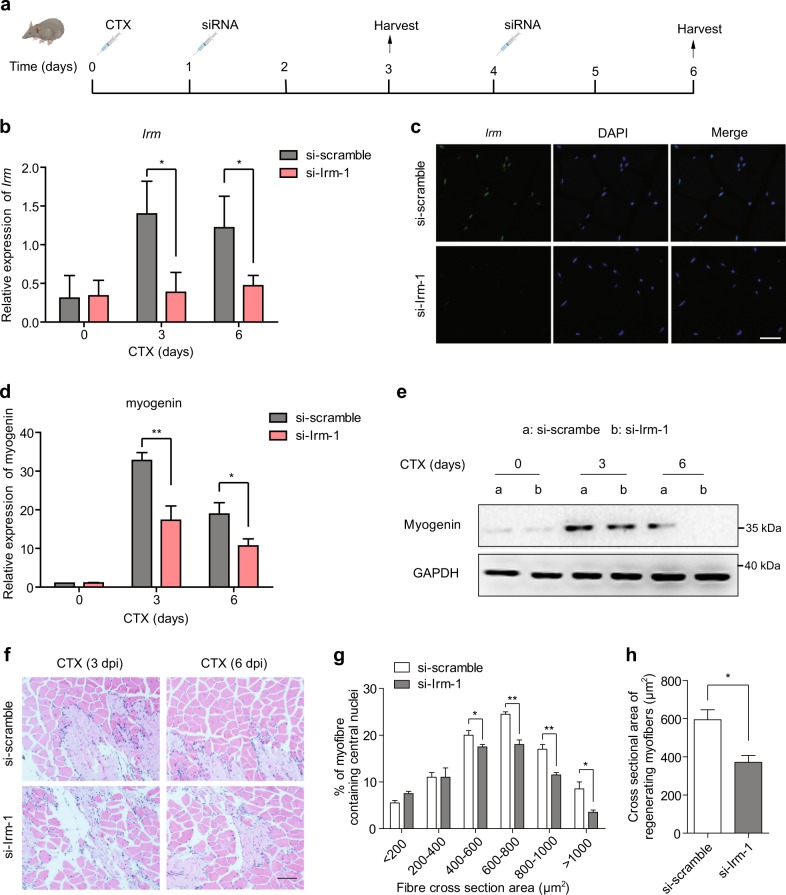
Knockdown of *Irm* inhibits injury-induced muscle regeneration in vivo. **a** Injection scheme for si-scramble or si-*Irm* into CTX-injured muscles. *n* = 8 mice for each group. **b** Relative expression levels of *Irm* at multiple time points after CTX injection, as detected by qRT-PCR. **c** RNA-FISH for detecting *Irm* 6 days post-CTX injury in muscle tissues. Green: *Irm*. Scale bar, 50 μm. **d**, **e** Knockdown of *Irm* reduced the mRNA (**d**) and protein (**e**) levels of myogenin at multiple time points after CTX injection. Myogenin protein levels were normalized to the GAPDH protein levels. **f** Representative hematoxylin and eosin (H&E)-stained sections of TA muscle 3 or 6 days post-CTX injury (3 or 6 dpi) induced by a CTX injection. Scale bars, 100 μm. **g** The cross-sectional areas of regenerating fibers on day 6 post-CTX injury using ImageJ software. Only myofibers containing centralized nuclei were measured. **h** The average area of the cross-sections of regenerating fibers on day 6 post-CTX injury; approximately eight random fields were captured, and > 1500 fibers were measured per sample. Data shown represent the mean ± SEM. ^*^P < 0.05, ^**^P < 0.05, and ^***^P < 0.05 by Student’s *t* test. CTX, cardiotoxin; qRT-PCR, quantitative real-time polymerase chain reaction; RNA-FISH, RNA fluorescence in situ hybridization; TA, tibialis anterior

The above findings underscored the role of *Irm* in myoblast differentiation, suggesting that it may play a role in muscle regeneration in vivo. To this end, we depleted *Irm* in mouse limb muscles during injury-induced regeneration. As illustrated in Fig. 4a, an injection of si-Irm-1 and si-scramble was performed two times on days 1 and 4 post CTX injection and muscles were harvested at the designated times for analyses. We found that the injection of si-Irm-1 led to a significant loss of *Irm* expression during the time course (Fig. 4b, c), as detected by RNA fluorescence in situ hybridization (RNA-FISH) and quantitative real-time polymerase chain reaction (qRT-PCR) assays. Consistently, the expression levels of myogenin were significantly decreased (Fig. 4d, e), as detected by western blotting and qRT-PCR assays. CTX induced extensive muscle damage in both si-Irm-1-group and si-scramble-group muscles on the day after injury (Fig. 4f). Regenerating myofibers, which are characterized by centralized nuclei, were significantly smaller in si-Irm-1 groups than in si-scramble groups at 6 days after CTX injection (Fig. 4f, g), with an average size of 371.5 ± 36.4 versus 594.8 ± 52.2 μm^2^ (si-Irm-1 versus si-scramble, *P* < 0.05; Fig. 4h). Altogether, these results demonstrated that the loss of *Irm* caused a significant delay in injury-induced muscle regeneration in vivo.

### *Irm* directly interacts with MEF2D

**Fig. 5:**
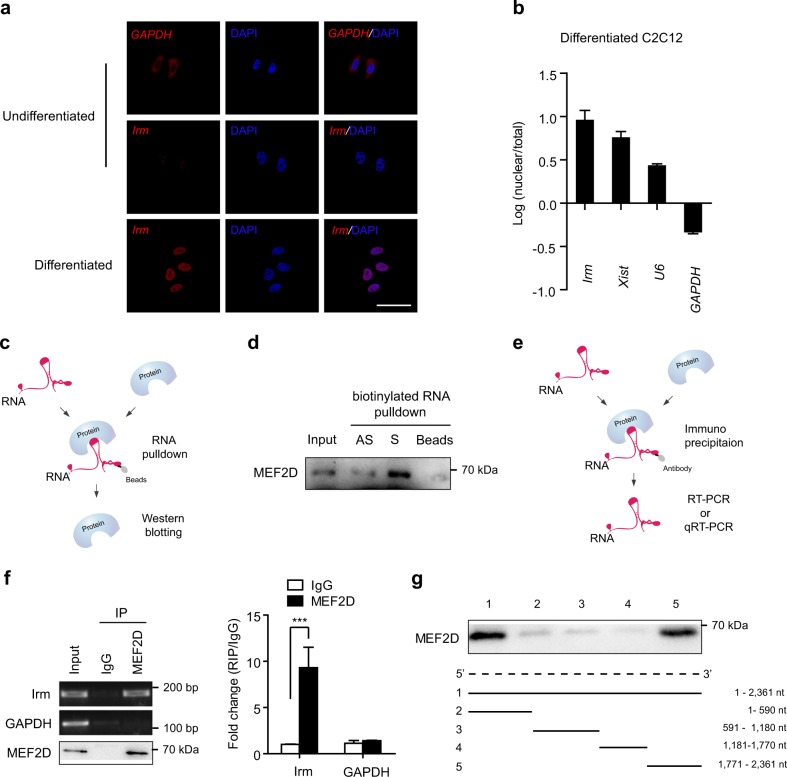
*Irm* directly binds to MEF2D. **a** RNA-FISH for detecting *Irm* and *GAPDH* in undifferentiated and differentiated C2C12 cells. Red: *Irm* or *GAPDH*. Blue: DAPI staining. Scale bar, 50 μm. **b** Relative abundance of *Irm* in total and nuclear RNAs of differentiating C2C12 cells, as detected by qRT-PCR. *U6*, *Xist*, and *GAPDH* were used as endogenous controls. **c** A schematic representation of RNA pull-down assay. **d** Western blotting assay for the specific interaction of *Irm* with MEF2D. **e** A schematic representation of RNA immunoprecipitation (RIP) assay. **f** RIP assay showed the association of MEF2D with *Irm* in vivo, as detected by RT-PCR and qRT-PCR. **g** Deletion mapping of the MEF2D-binding region(s) in *Irm*. Top: western blotting for MEF2D in protein samples pulled down by the different truncated *Irm* constructs. Bottom: diagrams of the full-length and truncated *Irms*. nt: nucleotide. Data shown represent the mean ± SEM of three independent experiments. n.s., not significant; ^***^P < 0.05 by Student’s *t* test. DAPI, 4′,6-diamidino-2-phenylindole; qRT-PCR, quantitative real-time polymerase chain reaction; RNA-FISH, RNA fluorescence in situ hybridization

Next, we probed into the molecular mechanisms underlying the promoting role of *Irm* in myogenesis. First, RNA-FISH assays in differentiating C2C12 cells showed that *Irm* was distributed in the nucleus, as distinct from the cytoplasmic transcript *GAPDH*, which was mainly found in the cytoplasm (Fig. 5a). Consistently, *Irm* was also found in nuclear extracts by performing cellular fractionation assay (Fig. 5b).

Many nuclear lncRNAs perform their functions through their interaction with proteins^18^. Therefore, to identify the proteins that are associated with *Irm*, biotin RNA pull-down assay was performed (Fig. 5c). The biotinylated *Irm* and biotinylated antisense *Irm* were transcribed in vitro and incubated with whole C2C12 cell lysates. Biotin-labeled transcripts and their associated cellular proteins were captured by streptavidin beads and then subjected to sodium dodecyl sulfate-polyacrylamide gel electrophoresis (SDS-PAGE) analysis. The same protein samples were then subjected to mass spectrometry analysis, and the proteins specifically binding to *Irm* were identified (Supplementary Table S2). Among them, MEF2D is a member of the MEF2 family of transcription factors, which act as key mediators of signal-dependent transcription in many different cell types to control developmental processes such as differentiation^19^. Therefore, we selected MEF2D for subsequent validation. First, the association of *Irm* with MEF2D was validated by immunoblotting assay, and the results showed that MEF2D was clearly detected in *Irm*-pull-down protein complexes, but not in the complexes associated with antisense *Irm* (Fig. 5d). To further validate this interaction between *Irm* and MEF2D, we carried out RNA immunoprecipitation (RIP) of C2C12 cell nuclear extracts (Fig. 5e). Reverse transcription-polymerase chain reaction (RT-PCR) and qRT-PCR analysis of antibody-enriched RNA revealed that MEF2D antibody pulled down significantly more *Irm* lncRNA than the IgG control (Fig. 5f).

Next, we sought to determine which region of the *Irm* transcript mediates its interaction with the MEF2D protein. We generated a series of *Irm*-deletion probes, which were biotinylated and utilized for RNA pull-down assay. We found that RNA probes containing 1771–2361 nts of the *Irm* gene, but not others, were capable of pulling down MEF2D, suggesting that the last 590 base pairs of *Irm* are necessary and sufficient to bind to MEF2D protein (Fig. 5g). Collectively, these findings showed that *Irm* directly interacts with MEF2D.

### *Irm* regulates MyoD/MEF2D transcriptional activity by binding to MEF2D

Myogenesis is determined by the master transcriptional regulatory factor MyoD (myogenic differentiation) in concert with other myogenic regulatory factors, such as the MEF2 family members^20^. Given that *Irm* directly interacts with MEF2D, we speculated that *Irm* may regulate the transcriptional activity of MyoD/MEF2D.

**Fig. 6:**
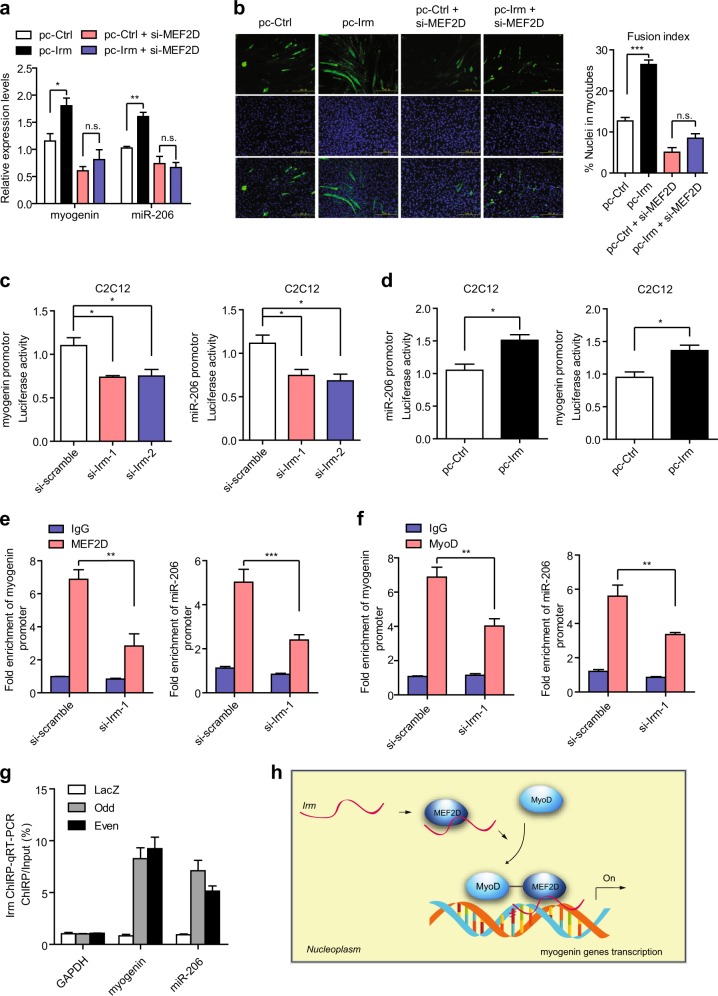
*Irm* enhances the transcriptional activity of MyoD/MEF2D. **a** The effect of *Irm* knockdown on the expression of myogenin and miR-206 depended on MEF2D, which was detected by qRT-PCR. **b** The effect of *Irm* knockdown on the differentiation of C2C12 cells depended on MEF2D. Fusion index was calculated. Scale bars, 50 mm. **c** C2C12 cells were transfected with si-Irm or si-scramble, and the luciferase reporter plasmids were generated by inserting the promoter region of myogenin or miR-206. The luciferase activities were measured 48 h after differentiation. **d** C2C12 cells were transfected with pc-Irm or pc-Ctrl, and the luciferase reporter plasmids were generated by inserting the promoter region of myogenin or miR-206. The luciferase activities were measured 48 h after differentiation. **e** Knockdown of *Irm* impaired the binding ability of MEF2D to myogenin and miR-206 promoters, which was determined by ChIP and qRT-PCR assays. **f** Knockdown of *Irm* impaired the binding ability of MyoD to myogenin and miR-206 promoters, which was determined by ChIP and qRT-PCR assays. **g** Chromatin isolation by RNA purification (ChIRP) assay was performed using even and odd antisense oligos tiling *Irm* and, a significant amount of genomic DNAs corresponding to myogenin and miR-206 promoters but not in glyceraldehyde 3-phosphate dehydrogenase (GAPDH) locus was retrieved. LacZ ChIRP retrieved no signal. **h** Model for *Irm*-regulating myogenesis. Data shown represent the mean ± SEM of three independent experiments. n.s., not significant; ^*^P < 0.05, ^**^P < 0.05, and ^***^P < 0.05 by Student’s *t* test. ChIP, chromatin immunoprecipitation; qRT-PCR, quantitative real-time polymerase chain reaction

To this end, we first investigated the effect of *Irm* on the expression of MyoD/MEF2D target genes. By performing qRT-PCR assay, we found that overexpression of *Irm* significantly increased the expression of myogenin and miR-206 (Fig. 6a), which are the targets of MyoD/MEF2D. Meanwhile, the knockdown of MEF2D significantly blocked *Irm*-induced myogenin and miR-206 upregulation in C2C12 cells (Fig. 6a and Supplementary Fig. S1). Consistently, the knockdown of MEF2D significantly inhibited the effect of *Irm* on the differentiation of C2C12 cells (Fig. 6b), suggesting that *Irm* regulates C2C12 cell differentiation and the expression of myogenin and miR-206 in an MEF2D-dependent manner. Next, we wanted to determine whether *Irm* plays a role in MyoD/MEF2D localization at the promoters of myogenin and miR-206. Luciferase reporter assays using myogenin and miR-206 luciferase reporters revealed inhibited activities with *Irm* reduction (Fig. 6c). Consistently, the overexpression of *Irm* had completely opposite effects in C2C12 cells (Fig. 6d). Chromatin immunoprecipitation (ChIP)-PCR assays revealed that *Irm* knockdown caused a significant reduction in the strength of MEF2D binding at the promoters of myogenin and miR-206 (Fig. 6e). Moreover, in the reduction of localization of MEF2D at the appropriate site, the association of MyoD at the promoter regions of myogenin and miR-206 was notably diminished (Fig. 6f). The chromatin isolation by RNA purification assay revealed that *Irm* directly bound to myogenin and miR -206 promoter regions (Fig. 6g). Taken together, these results strongly suggest that *Irm* may act as a scaffold lncRNA to recruit MyoD/MEF2D to the promoters of myogenin and miR-206 and thereby transactivate the transcription of myogenin and miR-206, leading to the initiatiation of myogenesis.

## Discussion

Skeletal myogenesis is tightly regulated by a complex network. Accumulating evidence suggests that lncRNAs play a significant role in the regulation of myogenesis^21^. Despite the rapidly increasing number of lncRNAs functionally investigated so far, only a few lncRNAs have been identified to play a role in myogenesis^22–25^. In this study, we reported that lncRNA *Irm* is upregulated during myogenesis. Functional analyses show that the overexpression of *Irm* enhances myogenic differentiation, whereas the inhibition of *Irm* had a completely opposite effect in vitro. Notably, the inhibition of *Irm* blocked damage-induced muscle regeneration in vivo. Mechanistically, *Irm* regulated the expression of myogenic genes by directly binding to MEF2D, which in turn promoted the assembly of MyoD/MEF2D on the regulatory elements of target genes. To the best of our knowledge, these findings supported that *Irm* is a novel player in MEF2D-regulating network and can promote C2C12 cell differentiation and myogenesis progression.

Recently, emerging reports are beginning to identify lncRNAs that regulate myogenesis, such as *H19*^26^, *BC1*^27^, and *MYOSLID*^28^. LncRNA *Irm* was previously identified as an alternatively splicing isoform of *Rian* gene^4^. Worth to note, a recent study has suggested that *Irm* displayed increased expression in tongue and skeletal muscles during embryonic development^17^, indicating that *Irm* may play a pivotal role in skeletal myogenesis. Indeed, the expression of *Irm* was significantly increased in differentiated muscle cells, indicating that *Irm* is associated with active myogenesis. Furthermore, our results showed that *Irm* significantly promotes the differentiation of C2C12 cells by performing gain or loss functional assays. Consistently, the loss of *Irm* caused a significant delay in injury-induced muscle regeneration in vivo. Our study provides a comprehensive characterization of *Irm* functionally. These findings demonstrated its important regulatory function in skeletal myogenesis.

MyoD and MEF2 families of transcription factors play a prominent role at virtually every step in the skeletal muscle development pathway^10, 29^. These two classes of transcription factors interact directly to establish a unique transcriptional regulation network for skeletal muscle gene activation and also to regulate the expression of one another in mutually reinforcing regulatory circuits^30^. Several lncRNAs with enhancer functions in the regulation of transcriptional factor activity have been reported, including *PR-lncRNA-1*^31^, *Evf-2*^32^, *MEG3*^33^, and enhancer RNAs^34^. Interestingly, the expression of MyoD/MEF2D target genes was significantly increased by *Irm* in C2C12 cells, suggesting that *Irm* may enhance the transcriptional activity of MyoD/MEF2D. Furthermore, ChIP assay showed that the knockdown of *Irm* significantly inhibited the binding of MyoD/MEF2D to the promoters of their target genes. Mechanistically, *Irm* directly binds to MEF2D and the DNA of the promoter region to recruit MyoD/MEF2D to cause the known target gene activation. Our data support the notion that *Irm* acts as an lncRNA enhancer of MyoD/MEF2D and synergistically regulates the transcription of myogenic genes in concert with MyoD/MEF2D to mediate myogenic differentiation.

In reviewing the results of this study, a potential limitation should be kept in mind. Although our results have shown that the effects of *Irm* on C2C12 cell differentiation in vitro depend on MEF2D, whether the effects of *Irm* on muscle regeneration in vivo depend on MEF2D is still unclear. It has been proved that MEF2D plays essential roles in muscle differentiation^35, 36^. However, a recent study has shown that the loss of MEF2D in mice has no effect on muscle regeneration in response to CTX injury^37^. These evidences suggested that the function of *Irm*/MEF2D axis in muscle regeneration in vivo is complex and confused, which deserves further investigation in future.

In summary, our results provide a molecular explanation for the *Irm*-mediated enhancement of MyoD/MEF2D transcriptional activity in myogenic differentiation. Moreover, our findings conceivably indicated that *Irm*, as a novel regulator of myogenic differentiation, might functionally act as an lncRNA co-activator in guarding the specificity of MyoD/MEF2D transcriptional activity during development.

## Materials and methods

### Cell lines

Mouse C2C12 myoblasts were cultured in DMEM supplemented with 10% fetal bovine serum (FBS), 2 mM L-glutamine, 100 U/ml penicillin, and 100 μg of streptomycin (1% Pen/Strep) at 37 °C in 5% CO_2_. For myogenic differentiation experiment, the cells were seeded in 60 mm plates, and on reaching 80~90% confluence, they were shifted to DMEM containing 2% horse serum.

### Primary myoblast isolation

Total hind limb muscles (eight 10-day-old mice) were digested with 0.25% type II collagenase (Life Technologies, Carlsbad, CA, USA) and 0.25% trypsin in 37 °C for 1 h. Furthermore, the fibroblasts were removed by differential centrifugation. Finally, the primary myoblast cells were certificated by desmin antibody (Santa Cruz Biotechnology, USA) and cultured in F10/DMEM medium (1:1) supplemented with 20% FBS and basic fibroblast growth factor (Life Technologies; 25 ng/ml).

### RNA extraction and qRT-PCR assay

Total RNAs were extracted from cultured cells or tissues using HiFi RNA Extract kit (Beijing BLKWbio Co., Ltd, China) according to the manufacturer’s protocol. The complementary DNAs were synthesized from 500 ng of total RNAs using PrimeScript RT reagent kit (Takara, Dalian, China) according to the manufacturer’s protocol. The qRT-PCR assays were performed in the BioRad IQ5 Real-Time PCR System (BioRad, Hercules, CA, USA) using KAPA SYBR^®^ FAST qPCR Master Mix (KAPA, Wilmington, UK). The relative expression of RNAs was calculated using the comparative Ct method. The primer sequences are described in Supplementary Table S1. Data represent the mean ± SEM of three independent experiments.

### Overexpression of *Irm*

For *Irm* overexpression, the *Irm* sequences were cloned into pcDNA3.1 vector (+) (Invitrogen, USA). The constructs were confirmed by DNA sequencing. The vectors with an intact sequence of *Irm* were transfected into mouse C2C12 myoblasts using Lipofectamine 2000 (11668–019; Invitrogen, USA), following the manufacturer’s instructions. All media contained 10% FBS, 100 U/ml penicillin, and 100 mg/ml streptomycin (15140–122; Gibco, USA). Cells were incubated in humidified incubators equilibrated with 5% CO_2_ at 37 °C.

### RNA interference

siRNAs against *Irm* were synthesized by GenePharma (Shanghai city, China). The sequences of siRNAs used against *Irm* were the following: siRNA-1, 5′-GCACACTACTGTTGAATTA-3′ and siRNA-2, 5′-GCAGGATTCAGATAGTAAT-3′. The sequence of siRNA used against *Mef2d* was siRNA, 5′-GAGGCAAAGGGTTAATGCATCATTT-3′. For siRNA transfection, the cells were transfected with the indicated siRNAs using Lipofectamine 2000 (11668–019; Invitrogen, USA), following the manufacturer’s instructions.

### Immunofluorescence assay

C2C12 cells were washed twice in 1 × phosphate-buffered saline (PBS), fixed with 4% paraformaldehyde for 30 min, permeabilized with 0.5% Triton X-100–PBS for 5 min, and then blocked for 30 min with 3% bovine serum albumin/0.2% Triton X-100–PBS. To detect endogenous MHC, cells were incubated with primary rat monoclonal anti-MHC (1:200; Santa Cruz Biotechnology, USA) for 12 h at 4 °C. Secondary antibodies were conjugated with Alexa488-labeled secondary antibody (Invitrogen, USA) and used in a 1:1000 dilution for 2 h at 37 °C. 4′,6-Diamidino-2-phenylindole was added for 5 min in the dark before taking images with a confocal microscope (ZEISS, LSM880 + ELYRAS.1). Eight micrographs per field were performed.

### Animal studies

C57BL/6 mice were purchased from Liaoning Changsheng Biological Technology Company and maintained in the Institute of Special Economic Animal and Plant Science of CAAS Institutions at a constant temperature and humidity with a standard diet. C57B/L mice were housed in animal care facilities for at least 15 days before experiment. Our works were approved by the Animal Care Committee of the Institute of Special Economic Animal and Plant Science of CAAS and were conducted in accordance with the CAAS Guide for the Care and Use of Laboratory Animals. For CTX (Latoxan, Valence, France) injection, a muscle injury was induced in mice by injecting CTX (50 ml of 10 μM CTX in PBS) into the right tibialis anterior muscles. Oligos were prepared by preincubating 20 μm siRNA oligos with Lipofectamine 2000 for 15 min and injections were made in a volume of 100 μl minimum essential medium (Invitrogen, NY, USA) on days 1 and 4. Eight mice were used in each group. The muscles were harvested on days 0, 3, and 6, and total RNAs and proteins were extracted for real-time RT-PCR and western blotting assays. Mice were killed by CO_2_, and all efforts were made to minimize suffering.

### Hematoxylin and eosin stainings

Fresh isolated tibialis anterior muscle samples were mounted on 4% paraformaldehyde. The sections of muscle tissues (10 μm) were subjected to hematoxylin and eosin staining using a commercial kit (Solarbio, China). The sections were detected using OLYMPUS Digital Pathology (OLYMPUS, Japan). Eight micrographs per field were performed for the photomicrographs.

### Western blotting analysis

For the protein analysis of whole-cell lysates, cells were lysed in radioimmunoprecipitation assay (CWBIO, China) buffer plus ethylenediaminetetraacetic acid-free protease inhibitor cocktail (04693132001; Roche). Total proteins (10–20 μg) were re-suspended in Laemmli buffer (63 mM Tris-HCl, 10% glycerol, 2% SDS, 0.0025% bromophenol blue (pH 6.8)) and electrophoresed on SDS-polyacrylamide gels. Then, the proteins were transferred to polyvinylidene difluoride membranes. Membranes were incubated with the indicated primary antibodies and anti-mouse or anti-rabbit secondary antibodies conjugated to horseradish peroxidase. The immunoreactive bands were detected using SuperSignal™ West Pico Chemiluminescent Substrate kit (Thermo Fisher Scientific, USA) and Western Blotting Detection System (BioRad, USA). To detect the protein levels of GAPDH, MHC, and myogenin, anti-GAPDH (1:5000; Santa Cruz Biotechnology, USA), anti-MHC (1:1000; Santa Cruz Biotechnology, USA), and anti-myogenin (1:2000; Santa Cruz Biotechnology, USA) antibodies were used.

### RNA fluorescence in situ hybridization

RNA-FISH analyses in C2C12 cells and muscle tissues were performed as described previously^38^. The RNA probes were prepared using the DIG RNA Labeling Kit (SP6/T7). PCR primers for RNA probe synthesis are the following: F, 5′-GCACACTACTGTTGAATTA-3′ and R, 5′-GCAGGATTCAGATAGTAAT-3′. The digoxigenin haptens were detected by fluorescein-conjugated anti-DIG Fab fragment (1:200; Roche, USA). The nuclei were stained with 4′,6-diamidino-2-phenylindole. The signals were observed under a confocal microscope (ZEISS, LSM880 + ELYRAS.1).

### Subcellular localization assay

RNAs were extracted from the nuclei and cytoplasm (total) or only nuclei (nuclear) of C2C12 cells using PARIS kit (Invitrogen) according to the manufacturer’s protocol. One microgram of RNA extracted from total and nuclear fractions, respectively, was used for qRT-PCR analyses of *Irm*, *Xist*, *U6* RNA (nuclear retained), and *GAPDH* mRNA (exported to the cytoplasm). Data represent the mean ± SEM of three independent experiments.

### RNA pull-down assay

RNA pull down was performed as previously described^39^. In brief, the pGEM-T vectors (Promega, Madison, WI, USA) carrying *Irm*, antisense of *Irm*, truncated fragments of *Irm* or GAPDH DNAs were linearized with the corresponding restriction enzymes to prepare the template DNAs for in vitro transcription. Then, biotin-labeled RNA transcripts were transcribed in vitro using T7 RNA polymerase (Roche, Basle, Switzerland). Three micrograms of in vitro biotinylated RNAs were mixed with proteins extracted from C2C12 cells, followed by targeting the RNAs with streptavidin beads (Millipore, Bedford, MA). The coprecipitated proteins were then visualized by western blotting.

### RNA immunoprecipitation assay

We performed RIP experiments using Magna RIP RNA-Binding Protein Immunoprecipitation Kit (Millipore, Bedford, MA, USA) according to the manufacturer’s instructions. The antibody against MEF2D (Santa Cruz Biotechnology, USA) was used for RIP. The coprecipitated RNAs were detected by qRT-PCR assay. Total RNAs (input controls) and isotype controls were assayed simultaneously to demonstrate that the detected signals were from RNAs specifically binding to MEF2D. Data represent the mean ± SEM of three independent experiments.

### Luciferase reporter assay

For luciferase reporter assay, reporter plasmids with the promoter region of myogenin or miR-206 were transfected into C2C12 cells by Lipofectamine 2000 (Invitrogen, USA) in 96-well plates. The luciferase activities were measured 48 h after differentiation by using Dual-Luciferase^®^ Reporter Assay System (Promega, Madison, WI, USA). Firefly activity was normalized to Renilla luciferase activity. Data represent the mean ± SEM of three independent experiments.

### Chromatin immunoprecipitation

MyoD and MEF2D ChIP was performed with Magna ChIP™ A/G One-Day Chromatin Immunoprecipitation Kits (Millipore, Bedford, MA) according to the manufacturer’s instructions. Briefly, the ChIPed DNA was eluted, reverse X-linked, purified, and analyzed by qRT-PCR. Data represent the mean ± SEM of three independent experiments.

### Chromatin isolation by RNA purification

Chromatin isolation by RNA purification was performed as previously described^40^. The antisense probes (20 nucleotides in length) were designed and divided into odd and even pools. Oligonucleotides were biotinylated at the 3′ end with an 18-carbon spacer arm. First, C2C12 cells, which were harvested, were cross-linked and sonicated. Cell lysates and the probes were then incubated at 37 °C for 6 h. Furthermore, magnetic streptavidin beads were added to pull-down probes and separated with a magnet strip. DNA isolated by phenol–chloroform–ethanol precipitation was used for qRT-PCR. DNA-binding enrichment was represented by the percentage of pull-down DNA over input DNA. Data represent the mean ± SEM of three independent experiments.

### Statistical analysis

All statistical analyses were performed using GraphPad Prism 5 software. All values are expressed as the mean ± SEM of three independent experiments. Student’s *t* test was used for statistical analyses. *P* values lower than 0.05 were considered to be statistically significant.

## Supplementary information

SUPPLEMENTAL MATERIAL
