# Supplementary material for: Long non-coding RNA Irm enhances myogenic differentiation by interacting with MEF2D
Source: Cell Death Dis. 2019 Feb 21;10(3):181. doi: 10.1038/s41419-019-1399-2 (PMC6385193; doi:10.1038/s41419-019-1399-2)
Supplement: Supplementary file 1 — SUPPLEMENTAL MATERIAL [file 41419_2019_1399_MOESM1_ESM.docx]

**Supplemental Material**

**Long non-coding RNA *Irm* enhances** **myogenic differentiation by interacting with MEF2D**

Running title: LncRNA-*Irm* Regulates myogenesis *via* MEF2D

Yutong Sui^1^, Yu Han^2^, Xingyu Zhao^2^, Dongsong Li^2*^, Guangyu Li^1*^

^1^State Key Laboratory for Molecular Biology of Special Economic Animals, Institute of Special Animal and Plant Sciences, Chinese Academy of Agricultural Sciences, Changchun, P. R. China

^2^Joint Surgery Department, No.1 Hospital of Jilin University, Changchun 130021, P.R. China

^*^Corresponding authors:

dongsong Li, PhD

130021 Changchun

Joint Surgery Department, No.1 Hospital of Jilin University, Changchun 130021, P.R. China

71 Xinmin Street

E-mail: [lidongsong@jlu.edu.cn](mailto:lidongsong@jlu.edu.cn)

Guangyu Li, PhD

130112 Changchun

Jilin Provincial Key Laboratory for Molecular Biology of Special Economic Animals, Institute of Special Animal and Plant Sciences, Chinese Academy of Agricultural Sciences

4899 Juye Street

E-mail: [liguangyu@caas.cn](mailto:liguangyu@caas.cn)

**Supplemental Figures**

**Figure S1. The protein levels of MEF2D in C2C12 cells expressing pcCtrl, pc-Irm or si-MEF2D.**

**
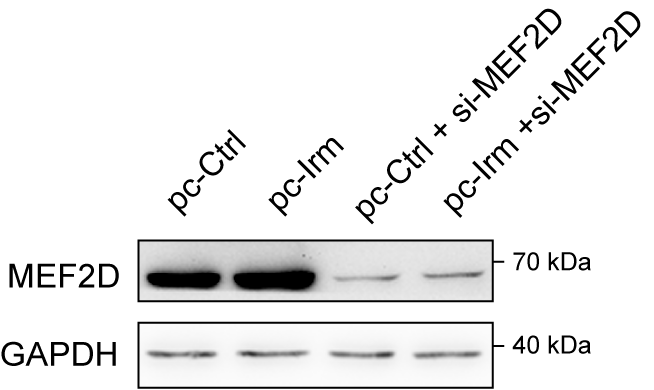
**

**Supplemental Table**

| **Table S1. Primers used for qRT-PCR or RT-PCR** | | |
| --- | --- | --- |
| **Genes** | **Forward primers sequence** | **Reverse primers sequence** |
| *Rian* | 5'-ACTGGTGGAAGTTGTTGT-3' | 5'-CAGAGGGTAGGTATTGAGT-3' |
| *Irm* | 5'-GTCACTAGAGGTCTGAGGTCCAT-3' | 5'-TCAATGCCTTAAATCCTTTCC-3' |
| *U6* | 5'-CTCGCTTCGGCAGCACA-3' | 5'-AACGCTTCACGAATTTGCGT-3' |
| *β-actin* | 5'-AGAGCCTCGCCTTTGCCGAT-3' | 5'-CCATCACGCCCTGGTGCCT-3' |
| *MyoD* | 5'-TATGATGACCCGTGTTTCG-3' | 5'-GCACCGCAGTAGGGAAGT-3' |
| *myogenin* | 5'-GCAATGCACTGGAGTTCGGT-3' | 5'-GCTGTCCACGATGGACGTAAG-3' |
| *MHC* | 5'-GATGGCACCGAAGTTGCTG-3' | 5'-TACTCATTGCCGACCTTGACC-3' |
| *GAPDH* | 5'-AATGTGTCCGTCGTGGATCTG-3' | 5'-TAGCCCAAGATGCCCTTCAGT-3' |
| *Xist* | 5'-GCCTCTTATTTGCGTGTA-3' | 5'-ACTTCTGAGCAGCCCTTA-3' |
| *myogenin promoter primers* | 5'-GAATCACATGTAATCCACTGGA-3' | 5'-ACGCCAACTGCTGGGTGCCA-3' |
| *miR-206 promoter primers* | 5'-GGAGTGATTGAGGTGGACAGA-3' | 5'-CTCTCCCACCTGTTTGTGTCTT-3' |
| *GAPDH promoter primers* | 5'-AAGCCAAACTAGCAGCTAGG-3' | 5'-GGGCTAGTCTATCATTGCAG-3' |

| **Table S2. Candidate associated proteins indentified in RNA Pull down/MS assays** | | | |
| --- | --- | --- | --- |
| **Genes** | **Descriptions** | **PSMs** | **Unique Peptides** |
| MEF2D | myocyte enhancer factor 2D | 54 | 44 |
| Hdx | Highly divergent homeobox | 23 | 17 |
| Tfe3 | Transcription factor E3 | 19 | 15 |
| Suz12 | Polycomb protein Suz12 | 16 | 9 |
| Mkrn1 | E3 ubiquitin-protein ligase makorin-1 | 11 | 8 |
| Carf | Calcium-responsive transcription factor | 11 | 8 |

MS, mass spectrometry; PSM, peptide spectrum match.

| **Table S3. The probes for ChIRP** | | |  |  |  |
| --- | --- | --- | --- | --- | --- |
| **Probe Number** | **Sequence (5' to 3')** | **Probe positon** | **Percent GC** | **Even** | **Odd** |
| 1 | gacttataatcccgaaacct | 2 | 40.00% |  | x |
| 2 | ctagtgacgtagtcatcgtc | 25 | 50.00% | x |  |
| 3 | ttctgctatggacctcagac | 47 | 50.00% |  | x |
| 4 | atgagagggtcacatgaggc | 70 | 55.00% | x |  |
| 5 | aaatggtgagagtgcatggc | 131 | 50.00% |  | x |
| 6 | tcttgattctgtgatctact | 154 | 35.00% | x |  |
| 7 | tttccgtgcatggagatttg | 176 | 45.00% |  | x |
| 8 | gttcaggattgatcaatgcc | 204 | 45.00% | x |  |
| 9 | gatttttcagtgtgtgcgta | 250 | 40.00% |  | x |
| 10 | acctttgtgtaagtcattct | 275 | 35.00% | x |  |
| 11 | catcatgaagacttcagcca | 337 | 45.00% |  | x |
| 12 | agcaggcaagacatcatgtg | 542 | 50.00% | x |  |
| 13 | catggtgagagtgcaaccac | 567 | 55.00% |  | x |
| 14 | agaatgcattttcagctgcc | 599 | 45.00% | x |  |
| 15 | cagctaggctgtgtaaatca | 629 | 45.00% |  | x |
| 16 | aggcatacattactgtgtct | 689 | 40.00% | x |  |
| 17 | atcctgcagacacaaacagg | 755 | 50.00% |  | x |
| 18 | cttgcaggatatccattact | 781 | 40.00% | x |  |
| 19 | caagggagactctacgaagc | 804 | 55.00% |  | x |
| 20 | agtgtcaatccacggacata | 849 | 45.00% | x |  |
| 21 | aatgaacttggggggaatcc | 874 | 50.00% |  | x |
| 22 | tacatcagcttaggggtatc | 955 | 45.00% | x |  |
| 23 | cacaaccaaggtgtacgcaa | 1038 | 50.00% |  | x |
| 24 | attcaacagtagtgtgcgtg | 1060 | 45.00% | x |  |
| 25 | catccatccacttggacaag | 1086 | 50.00% |  | x |
| 26 | aaagtgccaatcccattgag | 1108 | 45.00% | x |  |
| 27 | acagagtgcaggagcaatgg | 1130 | 55.00% |  | x |
| 28 | ccattgctgtgtgtacatta | 1169 | 40.00% | x |  |
| 29 | aggcatctgttccgaagaaa | 1219 | 45.00% |  | x |
| 30 | ctttgttggtgactatgcac | 1281 | 45.00% | x |  |
| 31 | cacccattgatgtgaagttg | 1303 | 45.00% |  | x |
| 32 | cacaatcaatcctggagctt | 1339 | 45.00% | x |  |
| 33 | ggttgtagctgtgactctaa | 1362 | 45.00% |  | x |
| 34 | agtcctcttgtgtctcgaag | 1457 | 50.00% | x |  |
| 35 | ctcagatcgcatcaagctta | 1525 | 45.00% |  | x |
| 36 | atagaattgtgtccagttgc | 1555 | 40.00% | x |  |
| 37 | tggagaaagcttctgggtta | 1580 | 45.00% |  | x |
| 38 | gtgtcactccatggtagaaa | 1631 | 45.00% | x |  |
| 39 | tgctgttaacggtagtttct | 1658 | 40.00% |  | x |
| 40 | ttgtattggaagtctgagcc | 1686 | 45.00% | x |  |
| 41 | gggtgaaaaagtacccagac | 1756 | 50.00% |  | x |
| 42 | agagatatttagacccagcg | 1829 | 45.00% | x |  |
| 43 | agagttcccagacactcaaa | 1930 | 45.00% |  | x |
| 44 | acaatatcaccaaccagacc | 1953 | 45.00% | x |  |
| 45 | ttttcaaccccataggaaga | 1976 | 40.00% |  | x |
| 46 | aaccactggactgagcatag | 2045 | 50.00% | x |  |
| 47 | cggagcctgacaaatgcaga | 2086 | 55.00% |  | x |
| 48 | tatagctatctccttagagg | 2112 | 40.00% | x |  |
